# Bayesian biclustering by dynamics: Algorithm testing, comparison against random agglomeration, and calculation of application specific prior information

**DOI:** 10.1016/j.mex.2020.100897

**Published:** 2020-04-22

**Authors:** Helen Pinto, Ian Gates, Xin Wang

**Affiliations:** a Geomatics Engineering, University of Calgary, 2500 University Drive NW, Calgary, AB, Canada, T2N 1N4; b Chemical and Petroleum Engineering, University of Calgary, 2500 University Drive NW, Calgary, AB, Canada, T2N 1N4

**Keywords:** Bbcd, Biclustering algorithm, Bayesian statistics, Steam-assisted gravity drainage (sagd) application

## Abstract

Bayesian Biclustering by Dynamics (BBCD) is a new clustering algorithm for Steam-Assisted Gravity Drainage (SAGD) oil recovery time series data [1]. In this companion paper the BBCD algorithm is tested on synthetic data, demonstrating use of the algorithm, as well as its robustness, and performance accuracy against Random Agglomeration. Supplementary information includes formulae to calculate analytical steam and oil volume data used as background knowledge for the SAGD application. Advantages of the BBCD algorithm are listed below.

• It includes background knowledge directly into the clustering process.
• It finds similarity between series and over time.
• It allows a user-specified definition for behaviour of interest, which relaxes dependency on series shape. This is important when similar behavioural events do not necessarily occur in the same temporal order.

## Graphical abstract

**Figure:**
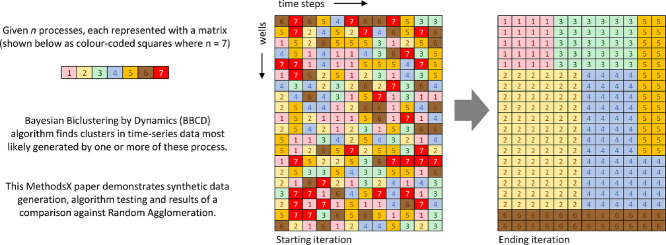

**Specifications Table**

**Table:** 

Subject Area	• Energy
	• Engineering
	• Computer Science
More specific subject area:	An algorithm that biclusters time-series data structured as Bayesian matrices, which makes it easier to interpret the resulting clusters.
Method name:	Bayesian Biclustering by Dynamics (BBCD)
Name and reference of original method	
Resource availability	Java software files, user guide, synthetic data and template files have been uploaded alongside this submission.

## Method details

The Bayesian Biclustering by Dynamics (BBCD) algorithm [1] is an extension of the Bayesian Clustering by Dynamics (BCD) algorithm [2], created to address the needs of a specific application in Steam Assisted Gravity Drainage (SAGD) oil recovery. The accompanying research paper [1] explains how this algorithm works with SAGD data, the results found, and a domain-specific interpretation of results. However, the BBCD algorithm itself is not application-specific. This paper describes synthetic data testing done to ensure that the BBCD algorithm is accurate and robust to noise. Results are compared against those of a Random Agglomeration algorithm.

Testing follows the approach taken in [3] where specific biclustered data sets are created to demonstrate algorithm performance under different testing scenarios. However, the process has been modified to create data sets that mimic typical SAGD data, except with known bicluster assignments. To understand the modifications, it is first necessary to understand the BBCD data structure. Therefore, the first section describes the BBCD algorithm and its data structures. The second section then describes the data generation process and why it differs from typical biclustering evaluation approaches. The third section lists the scenarios investigated, and finally the fourth section evaluates synthetic results and algorithm performance against Random Agglomeration.

## Bayesian biclustering by dynamics algorithm

**Fig. 1:**
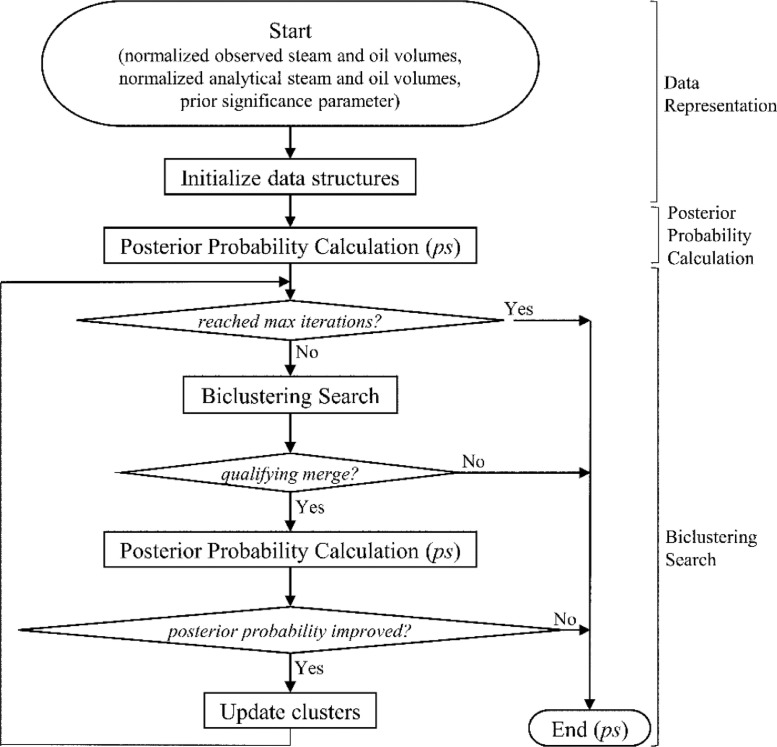
Flowchart of BBCD algorithm.

The Bayesian Biclustering by Dynamics is a greedy, agglomerative biclustering algorithm that automatically clusters both rows and columns of a data set into exclusive and exhaustive ‘checkerboard’ clusters. The algorithm also incorporates background knowledge directly into the clustering process via prior probability distributions. Clusters are described with transition probability matrices that capture the likelihood of transitioning from one discrete state at time *t-1* to another at time *t*. Fig. 1 shows a flowchart of how the algorithm works.

**Fig. 2:**
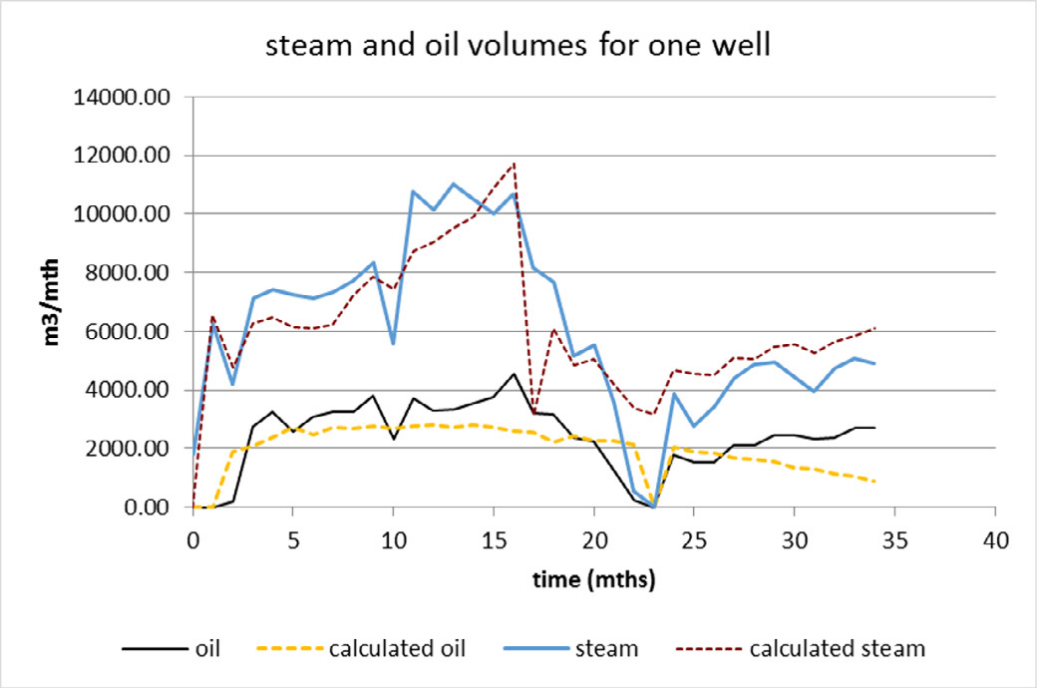
Observed and analytically calculated steam injection and oil production monthly volumes for one well *Note:* Further explanation on the appearance of these calculated series is given in supplementary material.

The algorithm starts with three inputs – a data set of observed values, a matched data set of prior values, and a significance level parameter that controls the influence of prior information on the clustering process. Prior information is background knowledge we have on a data set before the clustering process begins. In the accompanying research paper [1], it is noted that observed values consist of time series of historical steam injection and oil production monthly volumes for each SAGD well, whereas prior values consist of time series of analytical steam and oil volumes pre-calculated for each well. Therefore, four time series of equal length represent the life of one well, and different wells can have lifespans of different length. Fig. 2 shows the four time series for an example well that is 34 months old. For the analysis described in [1], 328 wells are used ranging between 19 – 104 months of age.

In overview, the BBCD algorithm proceeds as follows. First, the observed data set is initialized into node structures used by the search heuristic, and node structures used to calculate a posterior probability score. Nodes contain transition probability matrices that are the base data structure of the algorithm. A posterior probability score is then calculated for the initial configuration of nodes. Next. a biclustering search procedure uses a qualifying criterion to find the two most similar nodes to merge. The posterior probability of the new clustering configuration is calculated, and if the score has improved, the configuration is retained. The algorithm proceeds with iterative improvements until stopped by one of the following conditions: 1) a posterior probability score worse than the one found in the previous iteration, 2) a maximum iteration time out, 3) a merge-qualifying score exceeding a pre-specified cut-off factor, or 4) a single bicluster remaining.

### Data structures

For the BBCD algorithm to work, the input time series must first be discretized and converted into probability matrices. This data pre-processing step is necessary for two reasons: 1) to introduce a scale of ‘desirable’ or ‘undesirable’ behaviour between steam and oil volumes, and 2) to work with the posterior probability score equations. The descriptions that follow focus on the observed fluid volume sequences, but the same pre-processing steps are also applied to the analytically calculated sequences used as priors.

To create a set of discrete states, both steam and oil time sequenced volumes are first discretized into equal-frequency bins, for example: *low, medium* and *high*. Then, because each steam-oil pair of time series works in tandem, a combined state (*cstate*) is defined as a tuple of steam and oil states as shown in Table 1. The aim of SAGD is to achieve the highest oil recovery (oil = High) for the lowest steam injection (steam = Low), therefore *cstates* are ordered with values from 0 to 9. 1 and 9 are the least and most desirable *cstates* respectively, while a *cstate* value of zero represents months without steam injection or oil production. At the end of this step, one sequence of *cstates* has been created for each well.

**Table 1:** An example of steam-oil *cstates.*

steam	oil	*cstate*
either = 0		0
High	Low	1
Medium	Low	2
Low	Low	3
High	Medium	4
Medium	Medium	5
Low	Medium	6
High	High	7
Medium	High	8
Low	High	9

**Fig. 3:**
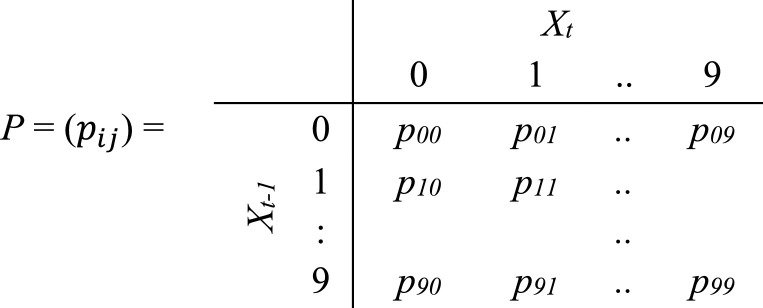
A matrix *P* of transition probabilities at time *t*.

Next, each sequence of *cstates* is converted into transition probability matrices. Let *x*$=\{x_{1},\;x_{2},\;\ldots ,\;x_{i},\;\ldots \}$ be a sequence of *cstates* representing the observed time series behaviour of well *X*, where each *x_i_* is one of the *cstates* 0 to 9. Then under the Markov assumption, the transition probability of *X* moving from *cstate i* at time *t-1* to *cstate j* at time *t* is $p_{ij}=\;p\left(x_{t}=j\;|\;x_{t-1}=i\right)$. *p_ij_* is calculated by first counting the transitions from *cstate i* to *cstate j* (*n_ij_*) in *X*, and then dividing by the total number of transitions originating from *cstate i* ($p_{ij}=\;n_{ij}\;/\;\sum_{j}\left(n_{ij}\right)$). Fig. 3 shows the resulting transition probability matrix *P*, where each row in *P* is a separate probability distribution summing to unity.

If *P* contains the probabilities of all transitions seen in well *X*, then it is the transition probability matrix of the *well-node* representing *X*. If *P* contains the probabilities of all transitions that occurred across all wells between time *t-1* and *t*, then it is the transition probability matrix of the *stage-node* representing time *t-1*. Finally, if *P* contains the probability of the single transition that occurred in well *X* between time *t-1* and *t*, then it is the transition probability matrix of the *segment-node* representing well *X* at stage *t-1*. All three types of node contain transition probability matrices, but well-nodes and stage-nodes are only used by the biclustering search procedure, whereas segment-nodes are used to calculate the posterior probability score. In effect, our dataset has been transformed into rows and columns of segment-nodes.

By defining wells and stages in this way, we are not concerned with the shape of the constituent time sequences, only the types of transitions that occurred. This is a less restrictive way of describing time sequences suitable for the SAGD application because wells may display similar behaviour but not always in the same order. Another argument against using shape-based time series algorithms is that monthly fluid volume curves are smoother in appearance than daily fluid volume curves, making it difficult to detect shape-based similarity between time sequences at the month aggregation level. This is not necessarily a disadvantage since SAGD field measurement of oil production is more accurate for monthly volumes than daily totals. Hence, any shape-based patterns found in daily volumes may be unreliable. Justification for the Markov assumption, which limits the BBCD algorithm to only search for one-step dependencies as opposed to multi-step dependencies, is that a lot can happen to a well in two months. For example, weak performance implied by a *cstate* transition of 3 → 3 in two consecutive months could indicate a well that is just starting up, or a well with underlying reservoir permeability limitations, or it could be due to a steam allocation shortage during those months. Given the uncertainty in interpreting one-step dependencies, multi-step dependencies would be even more difficult to interpret and justify. Note however, that one-step dependencies are a limitation imposed because of the SAGD application. The BBCD algorithm itself extends to applications with multi-step dependencies [1].

## Synthetic data generation

**Fig. 4:**
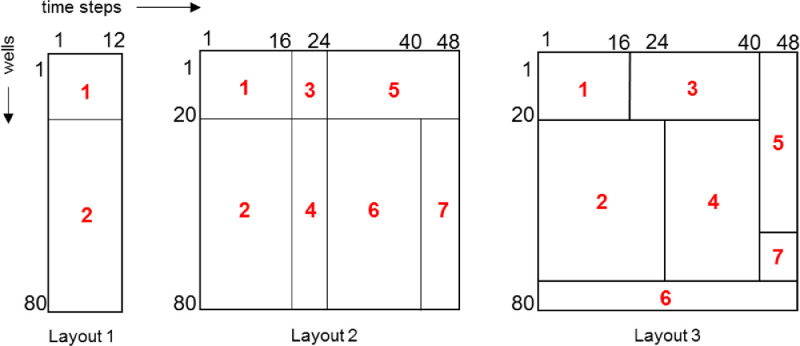
Layouts tested.

While the testing approach is similar to [3], the data sets themselves are created through a process described in [2]. A data set is built with a pre-defined arrangement of biclusters, each randomly assigned a number from 1 to *m* which represents the underlying process that bicluster was created by. Fig. 4 shows the three bicluster layouts created, with the underlying distributions used to generate their different sections shown in red. Layouts 1 and 3 are expected to be the most difficult to resolve because the former has very few time steps to work with, and the staggered clusters of the latter present a challenge to the search strategy. Time steps are assumed to be months, and therefore appear in multiples of 12.

Each underlying process is represented with a 10 × 10 transition probability matrix (MC) where the matrix rows represent *cstates* at time *t-1*, and columns represent *cstates* at time *t*. If there are *m* underlying processes represented as MC_1_ to *MC_m_*, then each bicluster is compared against all *m* MCs to find out which one it is closest to. Each bicluster is then assumed to have been generated by the MC with which is has the smallest Kullback-Liebler distance score [4]. The transition matrix for an underlying MC is created with a set of ten row distributions each with probability masses on 10 states (*cstates*). Each row distribution is sampled from a uniform, symmetric or skew-symmetric base distribution. To reduce selection bias, a set of sixteen row distributions is created, randomly drawn ten at a time (with repetition) to form each MC transition probability matrix. Defined in this way, the underlying MCs are not entirely distinct from each other. Table 2 shows matrix MC_1_ and the base distributions used to create each row.

**Table 2:** Transition probability matrix MC_1_*.*

**Distribution used to generate each row**	***MC_1_***										
	***cstates***	**0**	**1**	**2**	**3**	**4**	**5**	**6**	**7**	**8**	**9**
**left skewed**	**0**	0.031	0.060	0.169	0.308	0.357	0.041	0.001	0.001	0.001	0.001
**normal**	**1**	0.001	0.011	0.070	0.150	0.239	0.288	0.130	0.070	0.031	0.011
**uniform**	**2**	0.120	0.060	0.100	0.090	0.100	0.090	0.100	0.130	0.130	0.080
**right skewed**	**3**	0.001	0.001	0.001	0.001	0.001	0.100	0.704	0.140	0.051	0.001
**uniform**	**4**	0.120	0.060	0.100	0.090	0.100	0.090	0.100	0.130	0.130	0.080
**left skewed**	**5**	0.031	0.060	0.169	0.308	0.357	0.041	0.001	0.001	0.001	0.001
**normal**	**6**	0.011	0.060	0.100	0.140	0.179	0.150	0.130	0.130	0.070	0.021
**right skewed**	**7**	0.001	0.001	0.001	0.001	0.051	0.348	0.288	0.179	0.060	0.051
**right skewed**	**8**	0.001	0.001	0.001	0.001	0.001	0.100	0.704	0.140	0.051	0.001
**right skewed**	**9**	0.001	0.021	0.001	0.060	0.110	0.258	0.179	0.249	0.100	0.021

When generating data for the first well in Layout 2 shown in Fig. 4, *cstate* 3 (steam=Low, oil =Low) is selected at the first time point *t - 1*. The following *cstate* at time *t* is randomly selected from the row distribution for *cstate* 3 in *MC_1_*. Note that cumulative row distributions of MC_1_ are used to match randomly generated values to the corresponding *cstates*. Then, *cstate_t_* becomes the new $cstate_{t-1}$ and the process iterates until time point 16. At time point 16, the current $cstate_{t-1}$ is assumed to belong to generating matrix MC_3_ and the next *cstate_t_* is drawn from *MC_3_*. Similarly, at time point 24 the data generating matrix switches to MC_5_ and continues to time point 48. The process repeats until the first 20 synthetic wells are fully populated. The next 60 synthetic wells are generated in the same way, except that the underlying matrices involved are different. Starting at *cstate* 3, the first 16 transitions are chosen from MC_2_, the next eight transitions are chosen from MC_4_, followed by 16 from MC_6_ and the last eight from *MC_7_*. In all scenarios, the same MC matrices are also used to generate the corresponding prior data sets.

Before moving on, we explain why the bicluster-generating approaches mentioned in [3] (constant, constant upregulated, shift, scale and plaid) were not used. Typically, a synthetic bicluster is created first and contains either a constant value, or row entries shifted and/or scaled from some base row [5]. Then the bicluster is surrounded by background noise generated from a standard normal distribution. When each data element *x_rc_*, that is the *c*-th stage in well *X_r_*, is generated from a transition probability matrix, then typical bicluster-generating approaches will not work. For example, to generate a bicluster containing only the value 0, the underlying MC must have its top-left probability value representing *cstates* 0 → 0 set to 1, and every other value set to 0. This is not permissible since each row is considered a separate probability distribution that must sum to unity. Similarly, suppose $x_{rc}=7$ and needs to be shifted by 4. The result would be $x_{rc}=7+4=11$, but since only *cstate* values 0 – 9 are allowed the operation fails. Applying the modulo operation is equally problematic because the new value $x_{rc}=11\;mod\;10=1$ would entirely change the shape of the relevant row distribution. Scaling has the same problem as shifting, and the additive nature of plaid makes it unsuitable for creating checkerboard clusters that can be found simultaneously.

## Testing scenarios

Testing scenarios vary the parameters under which a test data set is generated. All the non-overlapping scenarios investigated in [3] are included, that is, the influence of noise, number of biclusters and size of biclusters. Additional layout and parameters choices specific to this application are also listed.

**Scenarios that test for algorithm robustness:**

a) Layouts used: 1, 2 and 3.
b) The length of each time series: 12 or 48 time-steps.
c) The number of time series generated: 80.
d) The number of different underlying distributions assumed to be present: 2, 4 or 7.
e) The prior significance level: *sig* = 0.01, 0.1, and 1.
f) The amount of noise introduced into each data set: 0%, 10%, 20% or 50%.
g) Number of bins (*cstates*) used for discretizing initial time series: 5, 10 or 17.
h) Merge qualifying criterion cut-off: 1, 2, 3 or 4.

Parameters in a), b) and c) are inherent in the layouts shown in Fig. 4. Synthetic data sets are generated as described in Section 2, using Layouts 1, 2 and 3. Each set contains 80 time series representing wells with either 12 or 48 time points. Set sizes are deliberately kept small so that the underlying *cstate* transition matrices are sparse, making it challenging for the BBCD algorithm to find useful similarity between clusters. Parameters in d) assume that a maximum of seven data-generating matrices (MCs) are used to generate the layouts. Parameters in e) and f) work together to see if giving higher significance to prior information has any effect on biclustering results as noise levels increase uniformly in both prior and observed data sets. Parameters in g) vary the initial number of *cstates* used in discretizing the time series to see the effect on accuracy of results. Parameters in h) test the sensitivity of results to the cut-off point. Beyond this point the merge-qualifying criterion is possibly merging clusters that should remain separate.

**Scenarios that test algorithm running time:**

a) Layout used: 2.
b) Scenarios generated: 80 × 48 (scale 1:1), 160 × 96 (scale 1:4), 320 × 192 (scale 1:16) and 640 × 384 (scale 1:64).

The four data sets contain the same seven biclusters in Layout 2 scaled up proportionally.

**Scenarios that test bicluster accuracy against random agglomeration:**

Scenarios a) to d) listed above are reused, but the choice of whether to merge two wells or two stages, and the specific wells (or stages) merged in each iteration, are chosen randomly.

## Evaluating synthetic results

**Fig. 5:**
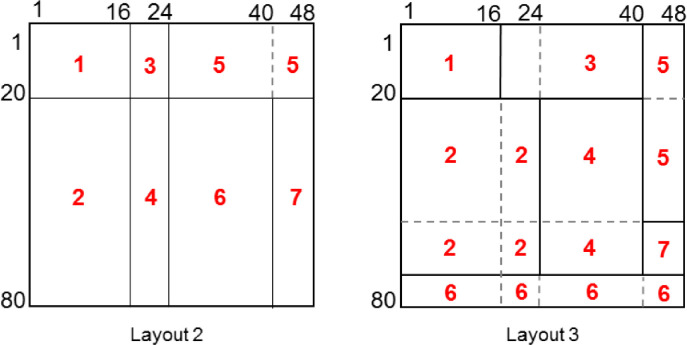
Ideal number of clusters found for Layout 2 and 3.

Since the search strategy can only find similarity between entire wells or entire stages, the number of clusters found will be greater or equal to the number present. For example, Layout 2 has seven clusters, but the best-case result will find eight clusters, two of which will be assigned to the same MC. Similarly, Layout 3 should find 16 clusters. Fig. 5 shows how this breakdown is calculated.

### Evaluation criterion

In evaluating the clusters, we count the number of *cstate* transitions correctly assigned in all biclusters (accuracy). *Cstate* transitions are counted if the cluster they belong to is assigned to the correct generating matrix *MC_i_*. The closest *MC_i_* matrix to each cluster is determined by Kullback-Liebler distance. This evaluation measure was proposed in [2] and works like the clustering error measure [6] modified in [3] for non-overlapping biclusterings.

### Algorithm robustness

Results in Table 3 show that accuracy is high even in the presence of noise but degrades as noise levels increase. When prior information contains as much noise as the observed data, results are less accurate especially when there are few time points to work with like in Layout 1. However, noise levels must be quite high (50%) to see this effect.

**Table 3:** Percentage accuracy of clustering results.

number of bins = 10, merge-qualifying cut-off = 2												
noise added to both observed and prior data sets	0%			10%			20%			50%		
*sig*	0.01	0.1	1	0.01	0.1	1	0.01	0.1	1	0.01	0.1	1
Layout 1	98	99	100	90	90	94	91	91	93	75	66	68
Layout 2	92	91	100	92	95	100	93	89	100	75	92	93
Layout 3	98	91	99	97	97	97	95	95	97	89	87	93

To examine the effect of bin size on accuracy of results, data sets are generated with 5, 10 and 17 states. Table 4 shows that the number of states matters most in very small data sets at high noise levels.

**Table 4:** Percentage accuracy with different initial binning.

*sig* = 1, merge-qualifying cut-off = 2												
noise	0%			10%			20%			50%		
bins	5	10	17	5	10	17	5	10	17	5	10	17
Layout 1	100	100	96	100	94	94	84	93	88	54	68	83
Layout 2	99	100	99	98	100	98	98	100	98	88	93	96
Layout 3	98	99	98	97	97	97	96	97	95	83	93	87

**Fig. 6:**
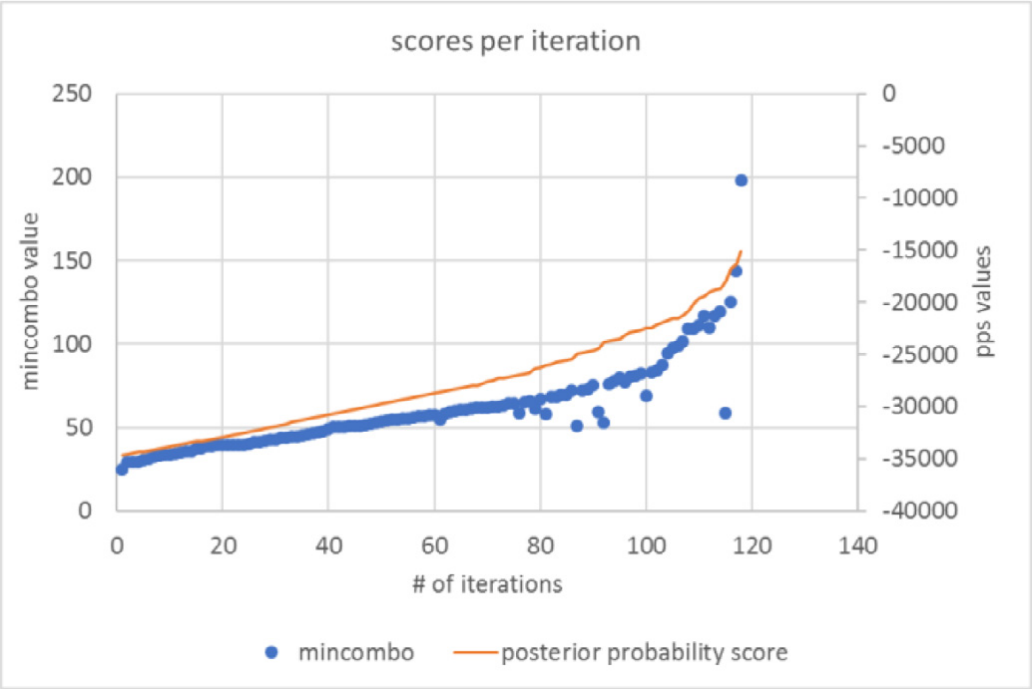
Posterior probability and *mincombo* scores for one test run.

Fig. 6 shows how the merge-qualifying score (*mincombo*) and posterior probability score rise as BBCD progresses. Both scores increase as the algorithm merges less similar clusters with each successive iteration, but it is easier to see where the score starts to rise exponentially in the trajectory of *mincombo*. We set a cut-off point to disqualify poor merges that tend to happen at the end of the iterative process. This stopping criterion is chosen via empirical observation as performance is tested at different cut-off points.

Table 5 shows the sensitivity of results to the merge-qualifying criterion cut-off point. A cut-off value of 1 is too low potentially stopping beneficial merges. Whereas a cut-off value of 3 or higher may be too permissive, allowing sub-optimal merges early in the clustering process

**Table 5:** Percentage accuracy associated with varying merge-qualifying cut-off points.

*sig* = 1, number of bins = 10, noise = 0%				
merge-qualifying cut-off	1	2	3	4
Layout 1	98	100	100	100
Layout 2	88	100	98	98
Layout 3	82	99	98	98

### Algorithm running time

**Fig. 7:**
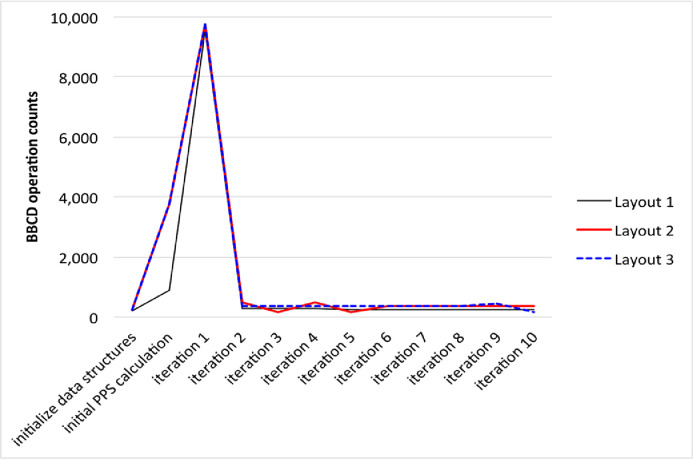
BBCD matrix operation counts.

Given that each cluster is represented by a 10 × 10 transition probability matrix, we count the number of matrix operations performed by the BBCD algorithm. Fig. 7 shows the number of matrix operations performed when initializing data structures, calculating the initial posterior probability score (PPS), and in each of the first ten iterations of runs using the three layouts. Computational load drops sharply after the first iteration because thereafter only clusters that merge need to be recalculated.

**Fig. 8:**
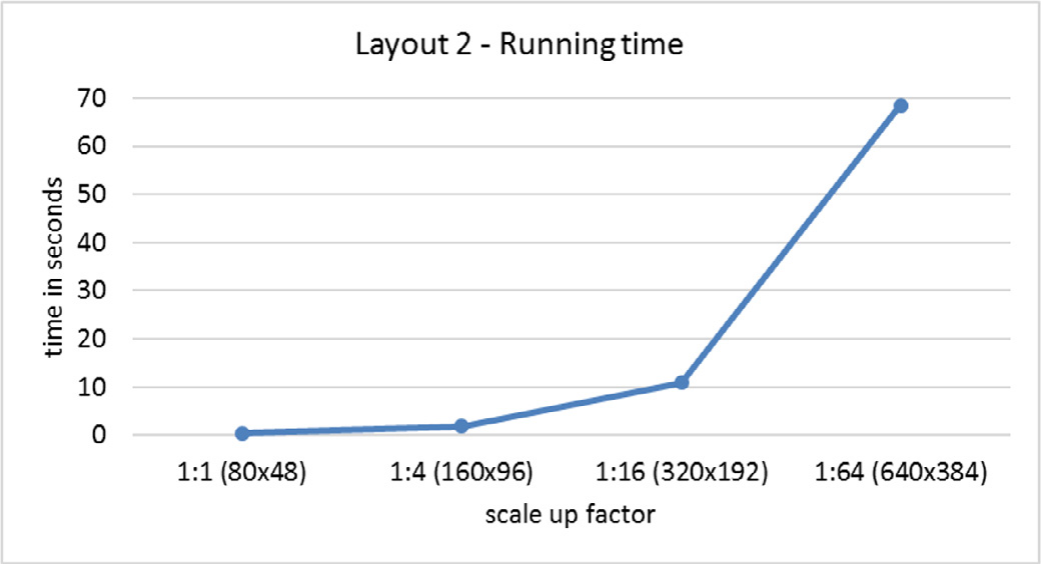
BBCD running time.

Fig. 8 shows a graph of the BBCD algorithm running time in seconds as Layout 2 is scaled up proportionally to create larger data sets. The algorithm has not been optimized for speed or efficiency.

### Algorithm accuracy against random agglomeration

Table 6 shows the results when the choices of whether to merge two wells or two stages, and the specific wells (or stages) to merge, are randomly selected. Two multi-class metrics are calculated – accuracy and the F1 score. Accuracy tracks the percentage of correctly deduced match-ups between *cstate* transitions and the MCs that generated them. The F1 score is the harmonic mean of two additional metrics (recall and precision). Recall tells us how many of the *cstate* transitions generated by a single MC (say MC_x_) have been identified; while precision tells us what proportion of *cstate* transitions attributed to MC_x_ are correctly assigned. An example of these multi-class metric calculations is shown in supplementary material.

**Table 6:** Comparison of BBCD against random agglomeration.

*sig* = 1, number of bins = 10, noise = 0%, merge-qualifying cut-off = 2				
	Accuracy		F1 Score	
	BBCD	Random agglomeration	BBCD	Random agglomeration
Layout 1	100	81	100	77
Layout 2	100	88	98	41
Layout 3	99	79	89	25

Results indicate that accuracy is surprisingly good with random agglomeration, but the F1 score shows that the approach has a poor balance between precision and recall when compared to BBCD.

## Appendix Supplementary materials

Image, application 1

Image, application 2

Image, application 3
