# Supplementary material for: Bayesian biclustering by dynamics: Algorithm testing, comparison against random agglomeration, and calculation of application specific prior information
Source: MethodsX. 2020 Apr 22;7:100897. doi: 10.1016/j.mex.2020.100897 (PMC7199012; doi:10.1016/j.mex.2020.100897)
Supplement: Supplementary file 2 [file mmc2.zip › user-guide.docx]

Bayesian Biclustering by Dynamics (BBCD)

User Guide

**Contents**

- Scope
- Background Information
  - Bayesian Biclustering by Dynamics algorithm
  - What are *cstates*?
  - Test data generation process
  - Evaluation measure
  - Evaluation results
- Downloading and Compiling
- Running the Program
- Validating the Results

**Scope**

The robustness of the Bayesian Biclustering by Dynamics (BBCD) algorithm is evaluated in a series of experiments run on three layouts of 80 records each (see Figure 2). Experimental testing scenarios vary in number of time steps, the number and location of clusters present (layouts), prior significance levels (*sig*), noise levels, and the number of initial bins used in discretization. Test results under these various scenarios can be found in [1]. This user-guide describes a subset of synthetic data testing done on the Bayesian Biclustering by Dynamics (BBCD) algorithm. Steam-Assisted Gravity Drainage (SAGD) data are not included due to a limited usage agreement with the provider, Divestco [2].

## **Background Information**

This section outlines the BBCD algorithm to better understand the input and output data. Next, the synthetic data generation process and three test layouts are explained, along with the evaluation measures and results.

Bayesian Biclustering by Dynamics algorithm

The Bayesian Biclustering by Dynamics (BBCD) algorithm automatically clusters both rows and columns of a data set, and then generates a descriptive summary for each cluster. Clusters are described with probability distributions that capture the likelihood of transitioning between discrete, consecutive states. BBCD incorporates background knowledge directly into the clustering process via prior probability distributions. The algorithm starts with three inputs – a data set of observed values, a matched data set of prior values, and a significance level parameter that controls the influence of prior information on the clustering process.

In the accompanying paper [3], observed values consists of historical steam injection and oil production volumes from SAGD wells, whereas prior values consist of analytical steam and oil volumes calculated for each well. Prior information is the background knowledge we have on a data set before the clustering process begins, representing a second set of discrete time series of the same size.

The BBCD algorithm proceeds as follows. First, the data sets are initialized into node structures used by the search heuristic, and node structures used to calculate the posterior probability score. Then the biclustering search procedure finds the two most similar nodes to join. The posterior probability of the new clustering configuration is calculated, and if the score has improved, the configuration is retained. The algorithm proceeds with iterative improvements until there are no more potential joins that satisfy a join-qualifying criterion. Figure 1 shows a flowchart of how this greedy, agglomerative clustering algorithm works.


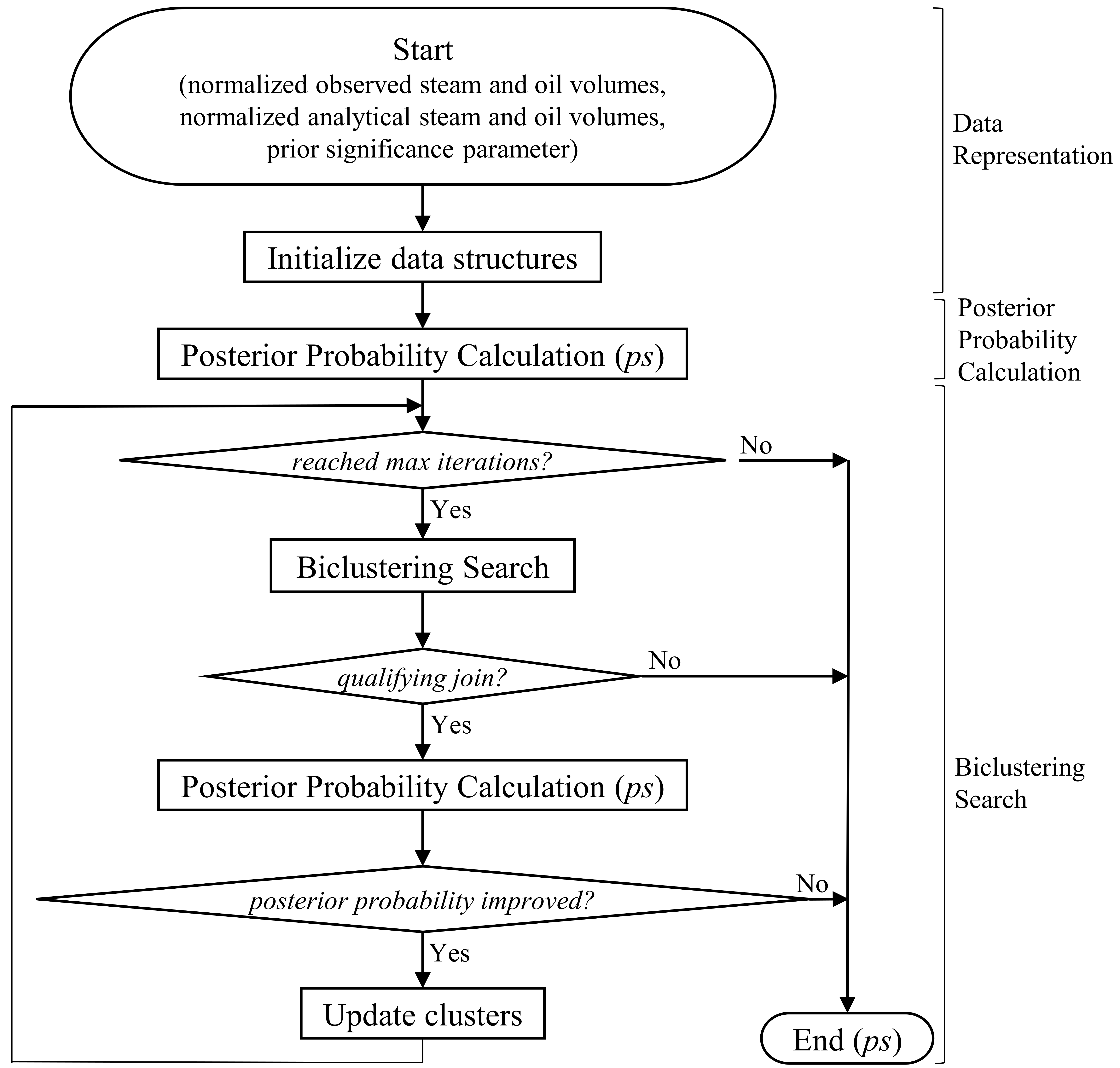


Figure 1: Flowchart of BBCD algorithm**.**

What are *cstates*?

Test data is designed to mimic discretized steam injection and oil production data from SAGD oil production wells. Both steam and oil volumes are first discretized into equal-frequency bins: *low*, *medium* and *high*. Discrete values are known as states. A combined state (*cstate*) contains a tuple of steam and oil states. Table 1 shows an example lookup table of *cstates* with values from 0 to 9.

Table 1: An example of steam-oil *cstates*

| steam | oil | *cstate* |
| --- | --- | --- |
| either = 0 | | 0 |
| High | Low | 1 |
| Medium | Low | 2 |
| Low | Low | 3 |
| High | Medium | 4 |
| Medium | Medium | 5 |
| Low | Medium | 6 |
| High | High | 7 |
| Medium | High | 8 |
| Low | High | 9 |

Test data generation process

Each test data set generated consists of a matrix of *cstates*. The rows of a data set contain time series representing SAGD well performance, while the columns represent months in the life of each well. Figure 2 shows three test scenarios varying in length of time series, and in the underlying matrices used to generate their different sections (shown in red).


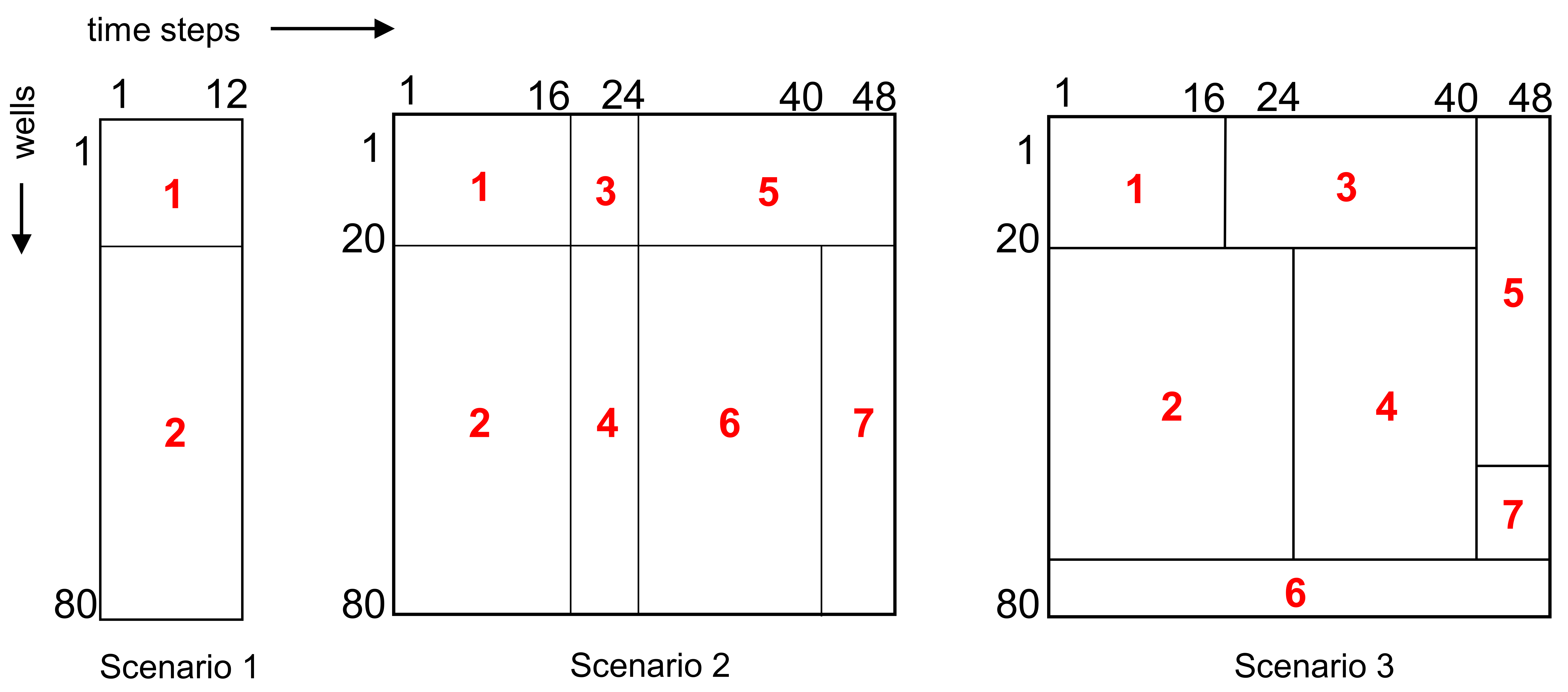


Figure 2: Layouts tested.

Since the search strategy can only join entire wells or entire stages, the number of clusters found will not always equal to the actual number present. For example, Scenario 2 has seven clusters, but the best-case result will find eight clusters, two of which will be assigned to the same *P* matrix. Similarly, Scenario 3 should find 16 clusters. Figure 3 shows how this breakdown is calculated.


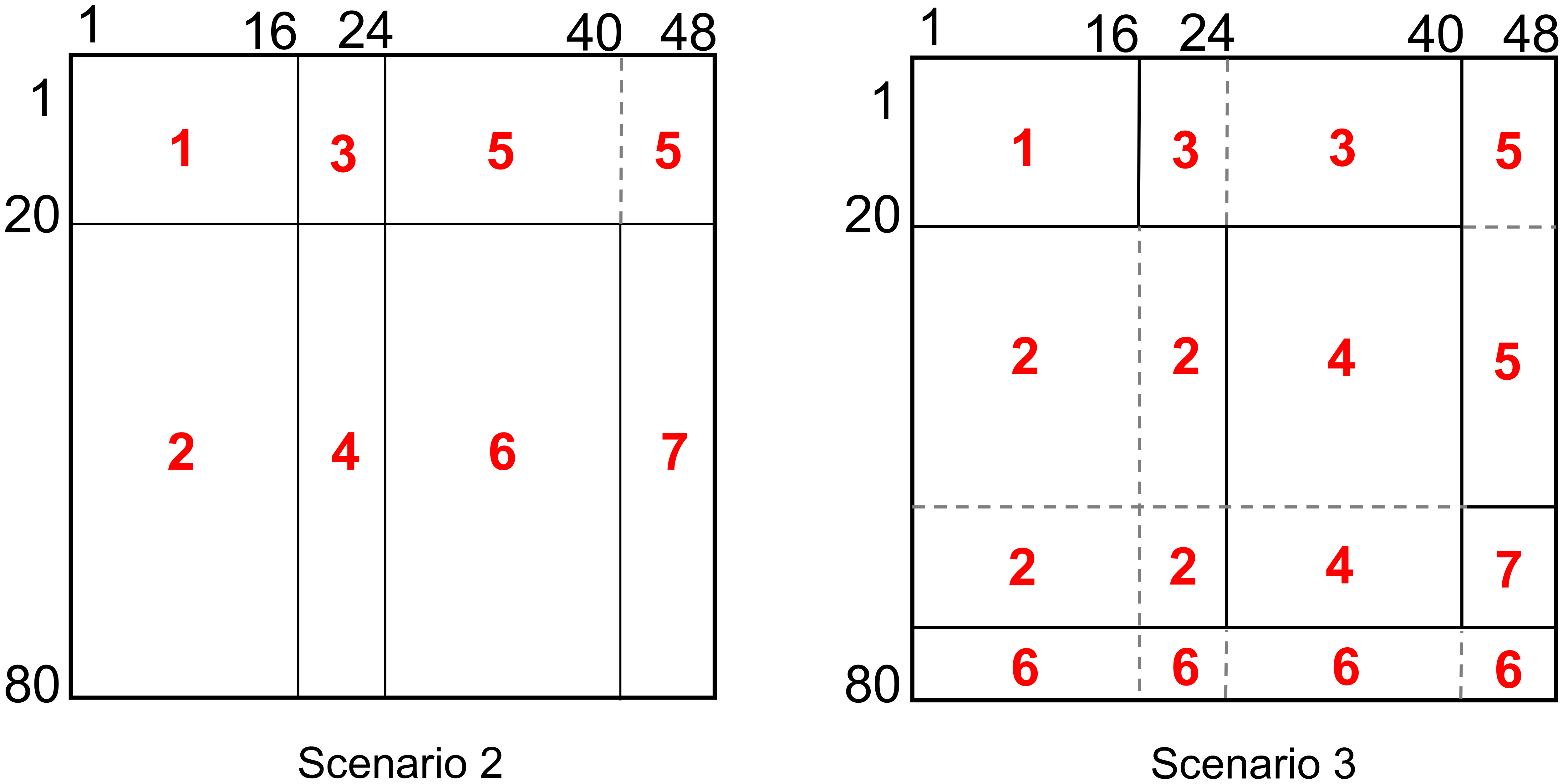


Figure 3: Ideal number of clusters found for Scenarios 2 and 3.

Evaluation measure

State transitions are counted if the cluster they belong to is assigned to the correct generating matrix, MC_x_. The closest MC_x_ matrix to each cluster is determined by Kullback-Liebler distance.

Evaluation results

On average, the clustered models improve the posterior probability of the data, which means that the algorithm recognized the presence of clusters within the test data sets. In the excerpt below from output file **out_Sc2.csv**, the posterior probability score improves from -30046.3 to -12959.3, and the final number of clusters is eight. *Mincombo* is the search heuristic score used to determine the next two clusters to join.

well or stage identifiers

posterior probability scores

type of join

*mincombo* score

------------ output\out_Sc2.csv ------------

initial score = -30046.315487

0 -- stage: prevscore = -30046.315487, newscore = -29972.355114 (19, 20, -0.323538) (3680)

number of clusters

1 -- well: prevscore = -29972.355114, newscore = -29929.707305 (70, 72, -0.311108) (3634)

2 -- well: prevscore = -29929.707305, newscore = -29888.328007 (33, 35, -0.309836) (3588)

…

119 -- well: prevscore = -13763.221230, newscore = -13380.870275 (0, 4, 5.114006) (12)

120 -- well: prevscore = -13380.870275, newscore = -12959.278218 (0, 1, 4.837339) (8)

final score = -12959.278218

The excerpt below from output file **out_Sc2_apr.csv** shows the accuracy, precision and recall measures calculated in each iteration.

------------ output\out_Sc2_apr.csv ------------

0: accuracy = 0.756991, precision = 0.188271, recall = 0.171548

1: accuracy = 0.757067, precision = 0.184559, recall = 0.171845

2: accuracy = 0.757067, precision = 0.177367, recall = 0.171845

…

119: accuracy = 1.000000, precision = 1.000000, recall = 1.000000

120: accuracy = 1.000000, precision = 1.000000, recall = 1.000000

# **Downloading and Compiling**

Download and unzip BBCD.zip. This zip file contains the following folders and files. The readme.txt file included in this download lists and explains each file.


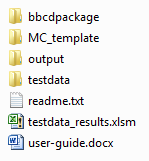


Figure 4: BBCD.zip file and folder structure.

NB: Due to a problem in uploading zip files, please note that after download and before compiling the code, the 13 supplementary files should be arranged within a folder entitled ‘BBCD’ as shown in Table 2:

Table 2: BBCD folder content

| Sub-folders | Files |
| --- | --- |
| bbcdpackage | BBCD.java  BBCDrun.java  Gamma.java |
| MC_template | MCs_7.csv  template_Sc2.csv |
| output | nmc_debug_Sc2.csv  out_Sc2_apr.csv  out_Sc2.csv |
| testdata | Sc2_prior.csv  Sc2.csv |
| - | readme.txt  testdata_results.xlsm  user-guide.docx |

Before compiling this code, make sure that Java JDK 1.8 or higher is installed. From a command line prompt, run the following Java command.

>> javac bbcdpackage/BBCDrun.java

# **Running the Program**

The BBCD algorithm requires nine input arguments listed below for the Sc2 test scenario:

1. testdata/**Sc2.csv** // represents observed data
2. testdata/**Sc2_prior.csv**  // represents background data
3. 1 // prior significance level (value between 0.01 and 1)
4. 2 // cutoff factor (1 to n)
5. 10 // represents a 10x10 *cstate* matrix
6. 10
7. output/**out_Sc2.csv** // output file
8. MC_template/**MCs_7.csv** // data-generating matrices used to estimate cluster assignments
9. MC_template/**template_Sc2.csv** // correct cluster assignments

From a command line prompt, run the following Java command:

>> java bbcdpackage/BBCDrun testdata/Sc2.csv testdata/Sc2_prior.csv 1 2 10 10 output/out_Sc2.csv MC_template/MCs_7.csv MC_template/template_Sc2.csv

# **Validating the Results**

Excel macro file **testdata_results.xlsm** is used to demonstrate the clustering process and show the most likely generating matrices for each cluster. Assume that the scenario Sc2 has just run.

1. Clustering process demonstration.

- Open **testdata_results.xlsm** and enable macros.
- Copy the entire contents of **out_Sc2.csv** to cell A3 on worksheet ‘Sc2’.
- Place cursor on cell AB2. Press *ctrl+d* to run drawClusters() macro.
- Result: The macro should automatically draw the cluster joins chosen in each iteration.

1. Matrix matching check.

- Open **testdata_results.xlsm** and enable macros.
- Open **nmc_debug_Sc2.csv** in Excel (first entry is on row 51).
- Copy the first 81 columns from nmc_debug.csv to cell A1 on worksheet ‘Sc2 APR’ of test_data_results.xlsm. Close nmc_debug.csv.
- Copy cells G5:I125 from worksheet ‘Sc2’, to cell CE5 on worksheet ‘Sc2 APR’. Make sure to copy values only.
- Place cursor on cell B51. Press *ctrl+e* to run evalMatrix() macro. Follow commented instructions in macro evalMatrix() before running.
- Result: The macro should display the nearest generating MC matrix to each of the 80x48 cells in Scenario 2 (see columns CI to FJ and corresponding rows). Accuracy, precision and recall measures are shown in columns FV to FX.

# **References**

[1] Pinto, H., Gates, I., Wang, X. (Submitted for publication). Bayesian Biclustering by Dynamics: Algorithm Testing, Comparison against Random Agglomeration, and Calculation of Application Specific Prior Information.

[2] Divestco, 2016, “EnerGISite,” Divestco, Calgary, Canada, accessed June 3,2016, https://energisite.divestco.com/idcWellLogs2010/Home.aspx

[3] Pinto, H., Gates, I., Wang, X. (2019). Bayesian Biclustering by Dynamics: A Clustering Algorithm for SAGD Time Series Data. *Computers & Geosciences*, 133, 104304. doi:10.1016/j.cageo.2019.07.008
