# Supplementary material for: Bayesian biclustering by dynamics: Algorithm testing, comparison against random agglomeration, and calculation of application specific prior information
Source: MethodsX. 2020 Apr 22;7:100897. doi: 10.1016/j.mex.2020.100897 (PMC7199012; doi:10.1016/j.mex.2020.100897)
Supplement: Supplementary file 3 [file mmc3.docx]

**Supplementary material:**

**Multi-class metric example**

In the first 20 wells of Layout 2, time steps 1 to 16 correspond to 20 × 15 *cstate* transitions generated by MC_1_. Time steps 17 to 24 correspond to 20 × 8 *cstate* transitions generated by MC_3_ (including the time step 16 **→**17 transition), and time steps 25 to 48 correspond to 20 × 24 *cstate* transitions generated by MC_5_. Similarly, in the next 60 wells of Layout 2, time steps 1 to 16 correspond to 60 × 15 *cstate* transitions generated by MC_2_, time steps 17 to 24 correspond to 60 × 8 *cstate* transitions generated by MC_4_, time steps 25 to 40 correspond to 60 × 16 *cstate* transitions generated by MC_6_, and time steps 40 to 48 correspond to 60 × 8 *cstate* transitions generated by MC_7_. These represent the actual or ‘true’ MC assignments.

Table S1 below shows the predicted MC assignments in a single iteration of the BBCD algorithm when using a synthetic data set generated from Layout 2. From this data we calculate the true positive (TP), true negative (TN), false positive (FP) and false negative (FN) statistics. A true positive is a *cstate* transition that is generated by some underlying process, MC_x_, and predicted as generated by MC_x_. A true negative is neither generated by MC_x_, nor predicted as generated by MC_x_. A false positive is not generated by MC_x_, but is predicted as generated by MC_x_. A false negative is generated by MC_x_, but is predicted as generated by another MC process.

Metrics are then calculated for each known MC grouping using the following equations:

$$accuracy= {(TP+TN)}/{(TP+TN+FP+FN)}$$

$$precision= \mathrm{TP}/{(TP+FP)}$$

$$recall= \mathrm{TP}/{(TP+FN)}$$

Precision values are averaged over all clusters to obtain precision_M_. Treating all clusters equally in this way is known as macro-averaging [15] and denoted with a subscript M. Recall values are also macro-averaged to obtain recall_M_. Finally, the F1 score is calculated as follows:

$$F1 score= {(\beta}^{2}+1){(\mathrm{precision}_{M} \times\mathrm{recall}_{M})}/{(\beta^{2})(\mathrm{precision}_{M}+\mathrm{recall}_{M})}$$

where β sets the weighting given to the precision and recall metrics. In this synthetic example, $\beta=1$ is chosen to give equal weight to both metrics because there is no reason to favour one over the other.

Table S1: Actual versus Predicted *cstate* assignments, statistics and metrics calculated in one iteration of the BBCD algorithm using Layout 2 (see Figure 4 in accompanying MethodsX paper for layouts).

|  | *sig* = 1, # of bins = 10, noise = 0%, merge-qualifying cut-off = 2 | | | | | | | | | | | | | | | | | | |
| --- | --- | --- | --- | --- | --- | --- | --- | --- | --- | --- | --- | --- | --- | --- | --- | --- | --- | --- | --- |
|  |  |  | Predicted | | | | | | |  |  |  |  |  |  |  |  |  |  |
|  |  |  | MC_1_ | MC_2_ | MC_3_ | MC_4_ | MC_5_ | MC_6_ | MC_7_ |  | TP | TN | FP | FN |  | A | P | R | F1 |
| Actual | 20×15 | MC_1_ | 300 | 0 | 0 | 0 | 0 | 0 | 0 |  | 300 | 3252 | 208 | 0 |  | 0.94 | 0.59 | 1.00 |  |
|  | 60×15 | MC_2_ | 0 | 900 | 0 | 0 | 0 | 0 | 0 |  | 900 | 2860 | 0 | 0 |  | 1.00 | 1.00 | 1.00 |  |
|  | 20×8 | MC_3_ | 0 | 0 | 0 | 0 | 160 | 0 | 0 |  | 0 | 3600 | 0 | 160 |  | 0.96 | 0.00 | 0.00 |  |
|  | 60×8 | MC_4_ | 184 | 0 | 0 | 280 | 16 | 0 | 0 |  | 280 | 3280 | 0 | 200 |  | 0.95 | 1.00 | 0.58 |  |
|  | 20×24 | MC_5_ | 0 | 0 | 0 | 0 | 480 | 0 | 0 |  | 480 | 2618 | 662 | 0 |  | 0.82 | 0.42 | 1.00 |  |
|  | 60×16 | MC_6_ | 24 | 0 | 0 | 0 | 450 | 370 | 116 |  | 370 | 2800 | 0 | 590 |  | 0.84 | 1.00 | 0.39 |  |
|  | 60×18 | MC_7_ | 0 | 0 | 0 | 0 | 36 | 0 | 444 |  | 444 | 3164 | 116 | 36 |  | 0.96 | 0.79 | 0.93 |  |
|  |  |  |  |  |  |  |  |  |  | Σ | 2774 | 21574 | 986 | 986 | avg | 0.93 | 0.69 | 0.70 | 0.69 |

**Analytical Steam and Oil Volume Calculations**

In this section the formulae used to calculate analytical oil and steam volumes are compiled. When this pre-analysis step is complete, analytical steam and oil volume sequences are created for each well pair in the observed data set. Figure 2 in the accompanying MethodsX paper shows an example of what these calculated time series look like. Note that while analytical volumes typically result in a smooth curve, each steam volume point shown in Figure 2 is a summation of multiple independently calculated heat consumption sources, and therefore that curve is not smooth. In particular, at the point of coalescence with neighbouring steam chambers, three additional ‘excess’ heat sources must be accounted for, resulting in a noticeable transition in month 17. These calculations have since been refined in [12], but are shown here as they were used for all 328 wells relevant to our accompanying analysis paper [13]. The dip at month 23 in the analytically calculated oil volume is the result of an inserted condition to set a calculated oil volume to zero when the corresponding observed oil volume recovered is zero. This choice allows us to identify field production shutdown events with *cstate* 0 in both observed and calculated series, across all wells in the data set.


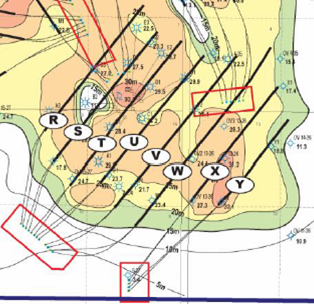


Figure S1: An example well pad from a report on the Alberta Energy Regulator (AER) website [10].

## **Required Data**

### **Reservoir Properties**

| OBIP | m3 | From AER reports – original bitumen in place. |
| --- | --- | --- |
| OBIP per well | m3 | Calculated by area, and by production over the first 24mths. |
| Permeability | D | From AER reports – specified for the well-pad. |
| Porosity | frac | From AER reports – specified for the well-pad. |
| Initial Oil saturation | frac | From AER reports – specified for the well-pad. |
| Residual oil saturation | frac | Set at 0.05 (estimate typically used in numerical simulation) |
| Pay thickness | m | Specified for the well-pad (calculated for each well-pair). |
| Initial reservoir temperature | °C | From AER reports – specified for the reservoir. |
| Initial reservoir pressure | kPa | From AER reports – specified for the reservoir. |
| Bitumen viscosity | cP | From AER reports – specified for the reservoir. |

### **Completions**

| Number of wells on pad |  |  |
| --- | --- | --- |
| Well-lengths | m | From AER reports or GeoCarta [9] – specified for the well-pad. |
| Inter-well spacing | m | From AER reports or GeoCarta [9] – specified for the well-pad. |
| Number of adjacent wells |  |  |

### **Temperature**

| Month-Year | date |  |
| --- | --- | --- |
| Time | sec |  |
| Injector temperature | °C | From AER reports – specified by well. |
| Producer temperature | °C | From AER reports – specified by well. |

### **Historical Fluid Volumes**

| Month-Year | date |  |
| --- | --- | --- |
| Oil volume | m3 | From GeoCarta [9]. |
| Steam volume | m3 | From GeoCarta [9]. |

### **General**

| Steam quality | % | From AER reports – specified by field or by pad. |
| --- | --- | --- |
| Steam temperature | °C | Calculated as average above 200°C on pad. |

### **Pre- Calculated Quantities**

| Oil viscosity at current temp | cP | Formula in [8] |
| --- | --- | --- |
| Water viscosity | cP | Formula in [8] |
| Permeability (effective) | m2 | Permeability(absolute) * permeability (relative) |
| Permeability (relative) | m2 | Adjusted to find least-squares fit. |
| Oil density | kg/m3 | Formula in [8] |
| Oil heat capacity | kJ/kgC | Formula in [8] |
| Water density | kg/m3 | Formula in [8] |
| Water heat capacity | kJ/kgC | Formula in [8] |
| Sandstone heat capacity | kJ/kgC | Formula in [8] |
| Overburden volumetric heat capacity | kJ/kgC | Formula in [8] |
| Reservoir volumetric heat capacity | kJ/kgC | Formula in [8] |
| Kinematic viscosity | m2/s | Formula in [8] |
| Reservoir thermal conductivity | W/mC | Formula in [8] |
| Thermal diffusivity | m2/s | Formula in [8] |
| Coefficient, m | - | Calculation from [3] |
| Calculated chamber height | m | Calculation from [3] |
| Calculated chamber half-width | m | Calculation from [14] |
| Time (dimensionless) | - | Calculation from [3] |
| Steam zone interface angle | rad | Calculation from [14] |
| Chamber height at overlap | m | Derived from first principles. |
| Area of overburden exposed to steam | m2 | Calculation from [6] |
| Chamber volume from cumoil | m3 | Calculation from [6] |
| Effective sweep efficiency | % | Calculation from [14] |
|  |  |  |
| Enthalpy of saturated vapour (H_V_) | kJ/kgC | Formula in [8] |
| Enthalpy of saturated liquid (H_L_) | kJ/kgC | Formula in [8] |

### **Analytical Oil Rate**

| Vertical growth of chamber (qgrowth) | m3 | Calculation from [4] |
| --- | --- | --- |
| Lateral expansion of chamber (qspread) | m3 | Calculation from [4] |
| Depletion model after coalescence (qdepl) | m3 | Calculation from [4] |
| qdepl (dimensionless) | - | Calculation from [4] |
| Combined oil rate | m3/d | min ( qgrowth , qspread , qdepl ) |

### **Analytical Steam Rate**

| E1 – heat required to expand the chamber | kJ | Calculation from [7] |
| --- | --- | --- |
| E2 – heat stored in reservoir ahead of chamber | kJ | Calculation from [14] |
| E3 – overburden heat loss during chamber rise | kJ | Calculation from [6, 7] |
| E3 – overburden heat loss during lateral expansion | kJ | Calculation from [6, 7] |
| E4 – heat loss factor | kJ |  |
|  |  |  |
| E1 – excess, after coalescence | kJ | Calculation from [11] |
| E2 – after coalescence | kJ | Derived from first principles. |
| E3 – excess, after coalescence | kJ | Calculation from [7, 11] |

### **Post- Calculated Quantities**

| Enthalpy (H_V_) reduced by steam quality | kJ/kgC | Calculation from [11, 14] |
| --- | --- | --- |
| Enthalpy (H_L_) reduced by produced condensate | kJ/kgC | Calculation from [11, 14] |
| ∆H | kJ/kgC | H_V_ – H_L_ |
| Steam injection rate - with chamber overlap (CWE) | m3 | CWE = cold water equivalent |
| Steam injection rate - without chamber overlap (CWE) | m3 | Calculation below. |

# **Approach for Calculating Oil Flow Rate**

The analytical calculation of oil flow rate used here follows Butler’s original approach [4]. However, any modified analytical approach such as [1, 2] can be substituted instead.

Excerpts from [6]:

*“The oil rate equation describing vertical growth of a steam chamber corresponds to early-stage development when steam injection has begun, and the chamber is beginning to develop, but has not yet reached the top of the reservoir. Since this expression is ever increasing, it is only valid to a maximum drainage rate predicted by the TANDRAIN or LINDRAIN assumptions* [4]*.”*

$$q=3L\left( \frac{k_{o}g\alpha}{m\nu_{s}} \right)^{2/3}\left( \varphi\Delta S_{o} \right)^{1/3}t^{1/3}$$

*“The drainage rate equation from the TANDRAIN or LINDRAIN assumptions, with the constants equal to 1.5 and 1.3 respectively, are valid for the mid-life of the well, after the steam chamber has reached the top of the reservoir and before steam chambers have coalesced with the adjacent steam chambers on a well pattern. The LINDRAIN result provides for a lower oil rate and appears to represent the behaviour of field SAGD performance better than that of the TANDRAIN model.”*

$$q=2L\sqrt{\frac{1.3\varphi{\Delta S}_{o}k_{o}g\alpha(h-y)}{m\nu_{s}}}$$

*“For the late-time phase of the life of a SAGD well-pair when the chambers are merging, the TANDRAIN or LINDRAIN depletion models can be applied to predict the reduction in drainage rate after the steam chambers on a well pattern have coalesced.”*

$$q=2L\left[ \frac{q^{*}}{\sqrt{\frac{\nu_{s}m}{k_{o}g\alpha\varphi\Delta S_{o}h}}} \right]$$

$$q^{*}=\frac{\sqrt{3}}{2}-\frac{\sqrt{2}}{3}\left( t^{*} \right)^{2}$$

$$t^{*}=\frac{t}{w}\sqrt{\frac{k_{o}g\alpha}{\phi S_{o}\nu_{s}\mathrm{mh}}}$$

*“Note that ∫q*dt* = 1 to ensure that the total amount of oil produced from the reservoir does not exceed the oil initially in place.”*

*“Since permeability is the variable with the highest uncertainty within the analytical model, it is used as the single adjustable parameter for matching the historical performance. As can be seen, the oil drainage rates for each phase overlap each other. The final oil drainage rate profile is obtained by taking the minimum of the predicted drainage rate for the three phases.”*


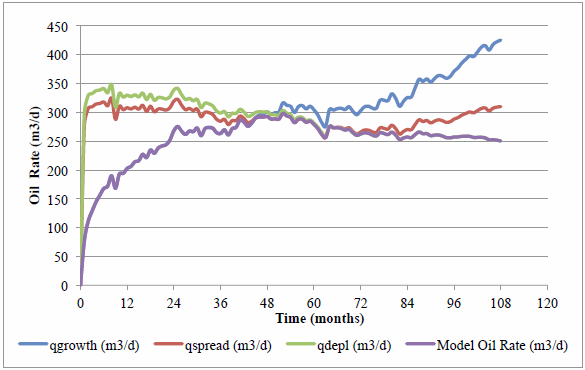


# Figure S2: Components of the oil drainage rate [6].

# **Approach for Calculating Steam Flow Rate**

Excerpts from [6]:

*“An analytical model has been developed to model heat consumption and distribution for [a] SAGD operation. It is based on the same idea as the original model proposed by Reis (1992) in which total heat requirements for the SAGD process were quantified for three main sources of heat consumption:*

- *Heat associated with expansion of the steam chamber.*
- *Heat stored in the reservoir ahead of the steam zone.*
- *Heat lost to the overburden.”*

*“Reis’ model was adapted by Edmunds and Peterson* [7] *to use an analytical expression for the overburden heat loss from Carslaw et al.* [5]*, as well as an empirically derived factor to account for heat stored below the moving front, and heat losses below the producer well.”*

*“Miura et al.* [11] *built on the Edmunds and Peterson’s work with several small revisions including:*

1. *A rising chamber phase from the expression derived by Butler* [3] *for chamber height.*
2. *Incorporation of subcool into the enthalpy calculations.”*

*“The final heat consumption model quantifies the heat consumption associated with the steam chamber expansion* [(E1)]*, the heat ahead of the steam zone* [(E2)]*, and the heat lost to the overburden* [(E3)]*. The heat loss to the underburden and due to convective losses has also been grouped and quantified as a single heat consumption term* [(E4)]*.”*

E1 – heat required to expand the chamber [7]:

Edmunds and Peterson model modified to allow top surface area, chamber height, and effective sweep efficiency to vary with time.

$$H_{c(t)}=\mathrm{Aob}_{(t)}\Delta TC_{\mathrm{vr}}h\eta_{s(t)}$$

E1 excess – after coalescence [11]:

Excess heat in chamber after coalescence.

$$H_{c excess (t)}=\left( \# of adjacent wells \right) \frac{1}{2}\left[ \mathrm{Aob}_{(t)}\Delta TC_{\mathrm{vr}}h\eta_{s(t)} \right]\left( \frac{t- t_{c}}{t} \right)^{2}$$

E2 – heat stored in reservoir ahead of chamber [14]:

Reis observed that this temperature distribution ahead of the steam chamber interface was constant regardless of position or time. This means that it is also independent of velocity. Therefore, the enthalpy ahead of the steam chamber interface proposed by Reis can be applied in this model even though it is developed using an exponentially decreasing velocity. This equation appears to be valid over the entire well life.

$$Q_{R1}=2L\frac{C_{\mathrm{vr}}\Delta Th}{\sin^{2}\theta}\sqrt{\frac{\varphi{\Delta S}_{o}mv_{s}h\alpha}{2ak_{o}g}}$$

E2 – heat stored in reservoir ahead of chamber after coalescence:

When chambers overlap, the length of the chamber interface reduces to h_x_.

$$Q_{R2}=nL\frac{C_{\mathrm{vr}}\Delta Th_{x}}{\sin^{2}\theta}\sqrt{\frac{\varphi{\Delta S}_{o}mv_{s}h_{x}\alpha}{2ak_{o}g}}+ \frac{(2-n)}{2}Q_{R1}$$

E3 – overburden heat loss during chamber rise [6, 7]:

The model assumes the incremental area exposed is not constant with time since the steam front velocity is an exponentially declining function. The integral is given by:

$$H_{o}=2\mathrm{Aob}_{t'}\Delta T\sqrt{\frac{k_{\mathrm{TH}}C_{\mathrm{vr}}}{\pi}}\int_{0}^{1} \sqrt{t' - \lambda(a)}da$$

$\int_{0}^{1} \sqrt{t' - \lambda(a)}da$ = $\int_{0}^{1} \sqrt{t^{'}+ \left[ \frac{\ln\left[ a\left( e^{-0.00000001245t^{'}}-1 \right)+1 \right]}{0.00000001245} \right]}da$

E3 – overburden heat loss during lateral expansion [6, 7]:

Heating the ceiling of the steam chamber when it is still within the reservoir is technically not considered a heat loss since the heat is being used to mobilize bitumen, however it is calculated as part of the overburden heat losses for the purposes of this study. In that regard, it is a pseudo heat loss. Once the chamber has reached the top of the reservoir, the volumetric heat capacity of the overburden is used in the heat loss calculation.

same formula as H_o_ above, except C_vr_ is replaced with C_vo_

E3 excess – after coalescence [1, 7]

This integration is used in the calculation of overburden *overlap* exposed to heat for different lengths of times. It works the same way as the previous numerical integration, except it starts after coalescence.

$$H_{o excess}= \left( \# of adjacent wells \right)\left[ \left( \mathrm{Aob}_{t'}\Delta T\sqrt{\frac{k_{\mathrm{TH}}C_{\mathrm{vo}}}{\pi}}. \int\left( t^{'}-t_{c} \right)- \lambda(a) \right)-\left( \mathrm{Aob}_{\mathrm{tc}}\Delta T\sqrt{\frac{k_{\mathrm{TH}}C_{\mathrm{vo}}}{\pi}}. \int\left( t'-t_{c} \right)- \lambda(a) \right) \right]$$

E_summation:

E_summation (before coalescence) = H_c_ + Q_R1_ + H_o_

E_summation (after coalescence) = (H_c_ – H_c excess_) + Q_R2_ + (H_o_ – H_o excess_)

Calculated steam injected CWE:

(E_summation * heat loss factor) / (∆H * ρ_w_)

**Nomenclature**

a = dimensionless temperature coefficient (a = 0.4 stated by Reis)

*a* = a dimensionless parameter which varies from 0 to 1 and represents fractions of the total heated area according to the time they are exposed

A = top surface area of the steam chamber

Aob_t_ = area of overburden exposed to steam as a function of time

C_vo_ = overburden volumetric heat capacity

C_vr_ = initial reservoir volumetric heat capacity

g = acceleration due to gravity

h = pay thickness

h_x_ = reduced pay thickness after coalescence (used to adjust length of chamber interface)

H_c_ = heat contained in the steam chamber

H_c excess_ = H_c_ overlap of adjacent chambers

H_L_ = enthalpy of saturated liquid

H_L’_ = enthalpy of produced condensate

H_o_ = heat lost to overburden

H_o excess_ = H_o_ overlap of adjacent chambers

H_V_ = enthalpy of saturated vapour

H_V’_ = enthalpy of saturated vapour reduced by steam quality

ΔH = H_V’_ - H_L’_

k_o_ = effective oil permeability

k_TH_ = reservoir thermal conductivity

L = well length

m = Butler’s viscosity coefficient

n = number of adjacent wells

q = flow rate

q* = dimensionless flow rate

Q_R1_ = total enthalpy in the formation ahead of the steam zone (before coalescence)

Q_R2_ = total enthalpy in the formation ahead of the steam zone (after coalescence)

S_o_ = oil saturation

t = time

t* = dimensionless time

t_c_ = time of coalescence

*t'* = time associated with a given heated area, Aob*_t’_* (N.B. *a* = 1 at *t'*)

T = temperature

v_s_ = kinematic viscosity at steam temperature

V_sz_ = steam zone volume

w = half the inter-well distance

y = vertical position of steam chamber

θ = angle of the steam/oil interface with respect to the horizontal plane

ϕ = porosity

α = thermal diffusivity

η_s_ = effective sweep efficiency = V_sz_/Ah

λ(*a*) = the time at which the area represented by *a* is exposed

ρ_w_ = water density

**References**

[1] Akin, S. (2006). Mathematical modeling of steam-assisted gravity drainage. *Computers & Geosciences*, 32, 240-246. doi:10.1016/j.cageo.2005.06.007

[2] Alali, N., Pishvaie, M., Jabbari, H. (2009). A new semi-analytical modeling of steam-assisted gravity drainage in heavy oil reservoirs. *Journal of Petroleum Science and Engineering*, 69, 261-270. doi:10.1016/j.petrol.2009.09.003

[3] Butler, R.M., (1998). Thermal Recovery of Oil and Bitumen, Gravdrain Inc.

[4] Butler, R.M., McNab, G.S., and Lo, H.Y. (1981). Theoretical Studies on the Gravity Drainage of Heavy Oil During In-Situ Steam Heating. Canadian Journal of Chemical Engineering, 59 (4). doi:10.1002/cjce.5450590407

[5] Carslaw, H.S., and Jaeger, J.C. (1959). Conduction of Heat in Solids, Oxford Univ. press.

[6] Chan, R. (2013). Application of Field Performance Data in Developing Simple Analytical Models to Predict the Performance of Steam Assisted Gravity Drainage. (Published master’s thesis). University of Calgary, Calgary, Alberta, Canada.

[7] Edmunds N., Peterson, J. (2007). A Unified Model for Prediction of CSOR in Steam-Based Bitumen Recovery, Canadian International Petroleum Conference, Calgary, Alberta, Canada, 12-14 June, Calgary, Alberta, Canada. doi:10.2118/2007-027

[8] Gates, I.D. (2011). Basic Reservoir Engineering. Kendall Hunt Publishing, Dubuque, IA.

[9] GeoCarta 3.0 for Data Analysis and Mapping. (2009). Divestco, last accessed July, 2017, http://www.divestco.com/software-and-data/geocarta-for-data-analysis/

[10] Japan Canada Oil Sands Limited (JACOS), Hangingstone SAGD 8788. (2016). Alberta Energy Regulator In-Situ Performance Presentations, accessed June 9, 2017, https://aer.ca/providing-information/data-and-reports/activity-and-data/in-situ-performance-presentations

[11] Miura, K., Wang, J. (2010). An Analytical Model to Predict Cumulative Steam Oil Ratio (CSOR) in Thermal Recovery SAGD Process. Canadian Unconventional Resources & International Petroleum Conference, 19-21 October, Calgary, Alberta. doi:10.2118/137604-MS

[12] Pinto, H. (2020). Insights on the Thermal Efficiency of SAGD from Data Analytics. Unpublished doctoral dissertation, University of Calgary, Calgary, Alberta, Canada.

[13] Pinto, H., Gates, I., Wang, X. (2019). Bayesian Biclustering by Dynamics: A Clustering Algorithm for SAGD Time Series Data. Computers & Geosciences, 133, 104304. doi:10.1016/j.cageo.2019.07.008

[14] Reis, J.C. (1992). A Steam-Assisted Gravity Drainage Model for Tar Sands: Linear Geometry. Journal of Canadian Petroleum Technology, 31(10). doi:10.2118/92-10-01

[15] Sokolova, M, Lapalme, G. (2009). A systematic analysis of performance measures for classification tasks. Information Processing and Management. 45: 427-437. doi:10.1016/j.ipm.2009.03.002
